# Supplementary material for: Human mobility and malaria risk in peri-urban and rural communities in the Peruvian Amazon
Source: PLoS Negl Trop Dis. 2025 Jan 6;19(1):e0012058. doi: 10.1371/journal.pntd.0012058 (PMC11737848; doi:10.1371/journal.pntd.0012058)
Supplement: S1 Table — (DOCX) [file pntd.0012058.s001.docx]

**Supplementary table 1: Data collection per month per community within each district.** Screenings performed by the P1 project (orange) and P3 project (blue).

| **Communities** | | **Data collection month** | | | | | | | | | |
| --- | --- | --- | --- | --- | --- | --- | --- | --- | --- | --- | --- |
|  |  | **Jul-18** | **Set-18** | **Oct-18** | **Nov-18** | **Abr-19** | **Jun-19** | **Set-19** | **Nov-19** | **Ene-20** | **Mar-20** |
| Iquitos | Tarapoto |  |  |  |  |  |  |  |  |  |  |
|  | Santa rita |  |  |  |  |  |  |  |  |  |  |
|  | Llanchama |  |  |  |  |  |  |  |  |  |  |
|  | San Jose de Lupuna |  |  |  |  |  |  |  |  |  |  |
|  | San Pedro |  |  |  |  |  |  |  |  |  |  |
| Mazan | Gamitanacocha |  |  |  |  |  |  |  |  |  |  |
|  | Libertad |  |  |  |  |  |  |  |  |  |  |
|  | Primero de Enero |  |  |  |  |  |  |  |  |  |  |
|  | Puerto Alegre |  |  |  |  |  |  |  |  |  |  |
|  | Salvador |  |  |  |  |  |  |  |  |  |  |
|  | Urco Mirano |  |  |  |  |  |  |  |  |  |  |
|  | Lago Yuracyacu |  |  |  |  |  |  |  |  |  |  |
|  | Huaman Urco |  |  |  |  |  |  |  |  |  |  |
