# Supplementary material for: Human mobility and malaria risk in peri-urban and rural communities in the Peruvian Amazon
Source: PLoS Negl Trop Dis. 2025 Jan 6;19(1):e0012058. doi: 10.1371/journal.pntd.0012058 (PMC11737848; doi:10.1371/journal.pntd.0012058)
Supplement: S2 Table — We did not have any recorded travel from satellite nodes (non assessed but recorded as destinations); thus, out degree was not computed. (DOCX) [file pntd.0012058.s002.docx]

**Supplementary table 2: Degrees and clusters per each node.** We did not have any recorded travel from satellite nodes (non-assessed but recorded as destinations); thus, out degree was not computed.

| **Communities** | **District** | **Degree** | | **W. degree** | **In Degree** | **W. In degree** | **Out degree** | **W. Out degree** | **Cluster** |
| --- | --- | --- | --- | --- | --- | --- | --- | --- | --- |
| Gamitanacocha | Mazan | 6 | 48 | | 1 | 1 | 5 | 47 | 1 |
| Libertad | Mazan | 22 | 152 | | 4 | 11 | 18 | 141 | 1 |
| Primero De Enero | Mazan | 9 | 47 | | 0 | 0 | 9 | 47 | 1 |
| Salvador | Mazan | 8 | 128 | | 0 | 0 | 8 | 128 | 1 |
| Lago Yuracyacu | Mazan | 11 | 73 | | 1 | 1 | 10 | 72 | 1 |
| Puerto Alegre | Mazan | 15 | 128 | | 0 | 0 | 15 | 128 | 1 |
| 14 de Julio | Mazan | 1 | 28 | | 1 | 28 | 0 | 0 | 1 |
| 22 de Noviembre | Mazan | 1 | 1 | | 1 | 1 | 0 | 0 | 1 |
| Curaray | Napo | 1 | 1 | | 1 | 1 | 0 | 0 | 1 |
| Hospital Regional | Iquitos | 1 | 1 | | 1 | 1 | 0 | 0 | 1 |
| Llachapa | Mazan | 1 | 2 | | 1 | 2 | 0 | 0 | 1 |
| Manco Capac | Indiana | 1 | 1 | | 1 | 1 | 0 | 0 | 1 |
| Mazan | Mazan | 8 | 463 | | 8 | 463 | 0 | 0 | 1 |
| Paucara Urco | Not identified | 1 | 1 | | 1 | 1 | 0 | 0 | 1 |
| Puerto Alegre Chacra | Mazan | 1 | 1 | | 1 | 1 | 0 | 0 | 1 |
| Putumayo | Not identified | 1 | 1 | | 1 | 1 | 0 | 0 | 1 |
| Quebrada Armas | Mazan | 2 | 7 | | 2 | 7 | 0 | 0 | 1 |
| Quebrada Calentura | Mazan | 1 | 2 | | 1 | 2 | 0 | 0 | 1 |
| Quebrada Chiriaco | Mazan | 2 | 2 | | 2 | 2 | 0 | 0 | 1 |
| Quebrada Chiriyacu | Mazan | 1 | 1 | | 1 | 1 | 0 | 0 | 1 |
| Quebrada Francisco | Mazan | 1 | 1 | | 1 | 1 | 0 | 0 | 1 |
| Quebrada Palometa | Mazan | 2 | 7 | | 2 | 7 | 0 | 0 | 1 |
| Quebrada Piuri | Mazan | 2 | 2 | | 2 | 2 | 0 | 0 | 1 |
| Quebrada Sara | Mazan | 1 | 1 | | 1 | 1 | 0 | 0 | 1 |
| Quebrada Tacsha Curaray | Napo | 1 | 1 | | 1 | 1 | 0 | 0 | 1 |
| Quebrada Tashamuena | Not identified | 1 | 4 | | 1 | 4 | 0 | 0 | 1 |
| Quebrada Tigre | Mazan | 1 | 1 | | 1 | 1 | 0 | 0 | 1 |
| Quebradapiure | Mazan | 1 | 1 | | 1 | 1 | 0 | 0 | 1 |
| Requena | Requena | 2 | 3 | | 2 | 3 | 0 | 0 | 1 |
| San Antonio | Mazan | 1 | 4 | | 1 | 4 | 0 | 0 | 1 |
| Santa Clotilde | Napo | 1 | 1 | | 1 | 1 | 0 | 0 | 1 |
| Santa Cruz | Mazan | 3 | 14 | | 3 | 14 | 0 | 0 | 1 |
| Santa Teresa | Not identified | 1 | 1 | | 1 | 1 | 0 | 0 | 1 |
| Tigre | Mazan | 1 | 2 | | 1 | 2 | 0 | 0 | 1 |
| Tipishca | Not identified | 1 | 2 | | 1 | 2 | 0 | 0 | 1 |
| Trompeteros | Trompeteros | 1 | 1 | | 1 | 1 | 0 | 0 | 1 |
| Trujillo | Not identified | 1 | 1 | | 1 | 1 | 0 | 0 | 1 |
| Yutococha | Mazan | 2 | 2 | | 2 | 2 | 0 | 0 | 1 |
| Zunicano | Indiana | 1 | 1 | | 1 | 1 | 0 | 0 | 1 |
| Urco Mirano | Mazan | 20 | 139 | | 2 | 2 | 18 | 137 | 2 |
| Huaman Urco | Mazan | 8 | 31 | | 0 | 0 | 8 | 31 | 2 |
| a.a.h.h Monte Sion | San Juan Bautista | 1 | 3 | | 1 | 3 | 0 | 0 | 2 |
| Antioquia | Trompeteros | 1 | 1 | | 1 | 1 | 0 | 0 | 2 |
| Buen Paso | Mazan | 1 | 1 | | 1 | 1 | 0 | 0 | 2 |
| Indiana | Indiana | 6 | 6 | | 6 | 6 | 0 | 0 | 2 |
| Nueva Vida | Napo | 1 | 2 | | 1 | 2 | 0 | 0 | 2 |
| Nuevo San Juan | Not identified | 1 | 1 | | 1 | 1 | 0 | 0 | 2 |
| Palmeras | Indiana | 1 | 2 | | 1 | 2 | 0 | 0 | 2 |
| Pucallpa | Calleria | 2 | 2 | | 2 | 2 | 0 | 0 | 2 |
| Pucayacu | Not identified | 1 | 1 | | 1 | 1 | 0 | 0 | 2 |
| Quebrada Ahuanari | Mazan | 2 | 2 | | 2 | 2 | 0 | 0 | 2 |
| San Antonio De Mirano | Mazan | 1 | 6 | | 1 | 6 | 0 | 0 | 2 |
| San Roman | Napo | 1 | 1 | | 1 | 1 | 0 | 0 | 2 |
| Santa Rosa | Mazan | 3 | 3 | | 3 | 3 | 0 | 0 | 2 |
| Tamanco | Mazan | 2 | 13 | | 2 | 13 | 0 | 0 | 2 |
| Tutapishco | Napo | 2 | 9 | | 2 | 9 | 0 | 0 | 2 |
| Union | Not identified | 1 | 1 | | 1 | 1 | 0 | 0 | 2 |
| Urbinas | Not identified | 1 | 1 | | 1 | 1 | 0 | 0 | 2 |
| Yanashi | Las Amazonas | 1 | 1 | | 1 | 1 | 0 | 0 | 2 |
| Tarapoto | Iquitos | 5 | 37 | | 0 | 0 | 5 | 37 | 3 |
| 31 De Agosto. Santo Tomas | San Juan Bautista | 1 | 1 | | 1 | 1 | 0 | 0 | 3 |
| Nuevo Milagro | San Juan Bautista | 1 | 2 | | 1 | 2 | 0 | 0 | 3 |
| Santa Clara | San Juan Bautista | 1 | 23 | | 1 | 23 | 0 | 0 | 3 |
| Santo Tomas | San Juan Bautista | 2 | 4 | | 2 | 4 | 0 | 0 | 3 |
| Santa Rita | Iquitos | 25 | 211 | | 1 | 5 | 24 | 206 | 4 |
| Llanchama | San Juan Bautista | 8 | 46 | | 0 | 0 | 8 | 46 | 4 |
| Lupuna | Iquitos | 7 | 67 | | 1 | 2 | 6 | 65 | 4 |
| San Pedro | Iquitos | 7 | 49 | | 0 | 0 | 7 | 49 | 4 |
| 3 unidos | Iquitos | 1 | 2 | | 1 | 2 | 0 | 0 | 4 |
| Atalaya | Alto Nanay | 1 | 1 | | 1 | 1 | 0 | 0 | 4 |
| Cacerio San Juan De Munich 9  (Rio Itaya) | Belen | 1 | 1 | | 1 | 1 | 0 | 0 | 4 |
| Cerro | Nauta | 1 | 12 | | 1 | 12 | 0 | 0 | 4 |
| Chacra | Iquitos | 1 | 1 | | 1 | 1 | 0 | 0 | 4 |
| Chambira | Not identified | 1 | 2 | | 1 | 2 | 0 | 0 | 4 |
| Comunidad Santa Maria Del Nanay | Alto Nanay | 1 | 1 | | 1 | 1 | 0 | 0 | 4 |
| Comunidad Tapira Por El Rio Marañon | Not identified | 1 | 1 | | 1 | 1 | 0 | 0 | 4 |
| Fray Martin | Iquitos | 1 | 4 | | 1 | 4 | 0 | 0 | 4 |
| Iquitos | Iquitos | 13 | 390 | | 13 | 390 | 0 | 0 | 4 |
| Jorge Chavez | Not identified | 1 | 1 | | 1 | 1 | 0 | 0 | 4 |
| Lima | Lima | 2 | 3 | | 2 | 3 | 0 | 0 | 4 |
| Manacamiri | Iquitos | 1 | 1 | | 1 | 1 | 0 | 0 | 4 |
| Nahuapa | Not identified | 1 | 1 | | 1 | 1 | 0 | 0 | 4 |
| Nauta | Nauta | 1 | 2 | | 1 | 2 | 0 | 0 | 4 |
| Nuevo Atalaya | Alto Nanay | 1 | 1 | | 1 | 1 | 0 | 0 | 4 |
| Nuevo Cusco, Rio Apayacu Cerca A Pebas | Las Amazonas | 1 | 1 | | 1 | 1 | 0 | 0 | 4 |
| Pacaya Samiria | Parinari | 1 | 1 | | 1 | 1 | 0 | 0 | 4 |
| Padre Coca | Punchana | 1 | 6 | | 1 | 6 | 0 | 0 | 4 |
| Quebrada De Nahuapa | Nauta | 1 | 1 | | 1 | 1 | 0 | 0 | 4 |
| Rio Momon. Gen Gen | Punchana | 1 | 1 | | 1 | 1 | 0 | 0 | 4 |
| Rumococha | San Juan Bautista | 2 | 3 | | 2 | 3 | 0 | 0 | 4 |
| San Juan | San Juan Bautista | 1 | 2 | | 1 | 2 | 0 | 0 | 4 |
| San Pablo | San Pablo | 1 | 2 | | 1 | 2 | 0 | 0 | 4 |
| Santa Maria | Alto Nanay | 1 | 1 | | 1 | 1 | 0 | 0 | 4 |
| Santa Victoria, Indiana | Indiana | 1 | 2 | | 1 | 2 | 0 | 0 | 4 |
| Tamshiyacu | Fernando Lores | 5 | 6 | | 5 | 6 | 0 | 0 | 4 |
| Tres Unidos | Iquitos | 2 | 7 | | 2 | 7 | 0 | 0 | 4 |
| Yarina | Iquitos | 1 | 1 | | 1 | 1 | 0 | 0 | 4 |
| Zungarococha | San Juan Bautista | 1 | 6 | | 1 | 6 | 0 | 0 | 4 |
