# Supplementary material for: Human mobility and malaria risk in peri-urban and rural communities in the Peruvian Amazon
Source: PLoS Negl Trop Dis. 2025 Jan 6;19(1):e0012058. doi: 10.1371/journal.pntd.0012058 (PMC11737848; doi:10.1371/journal.pntd.0012058)
Supplement: S3 Table — (DOCX) [file pntd.0012058.s003.docx]

**Supplementary table 3: Top ten destinations in the Overall Network (all communities), and per district network (Iquitos and Mazan)**

| **Full Network** | | **Iquitos-SJB Network** | | **Mazan Network** | |
| --- | --- | --- | --- | --- | --- |
| **Destination** | **W. In degree** | **Destination** | **W. In degree** | **Destination** | **W. In degree** |
| Mazan | 393 | Iquitos | 256 | Mazan | 463 |
| Iquitos | 349 | Santa Clara | 23 | Iquitos | 100 |
| 14 de julio | 28 | Cerro | 12 | 14 de julio | 28 |
| Santa Clara | 23 | Tres Unidos | 7 | Santa Cruz | 14 |
| Santa Cruz | 14 | Padre Coca | 6 | Tamanco | 13 |
| Libertad | 11 | Santa Rita | 5 | Libertad | 11 |
| Cerro | 9 | Fray Martin | 4 | Tutapishco | 9 |
| Tamanco | 9 | Tamshiyacu | 4 | Quebrada Armas | 7 |
| Tutapishco | 8 | Rumococha | 3 | Quebrada Palometa | 7 |
| Quebrada Armas | 7 | Santo Tomas | 3 | San Antonio de Mirano | 6 |
