# Supplementary material for: Human mobility and malaria risk in peri-urban and rural communities in the Peruvian Amazon
Source: PLoS Negl Trop Dis. 2025 Jan 6;19(1):e0012058. doi: 10.1371/journal.pntd.0012058 (PMC11737848; doi:10.1371/journal.pntd.0012058)
Supplement: S4 Table — (DOCX) [file pntd.0012058.s004.docx]

**Supplementary Table 4:** Complete list of relative importation scores for each pair of locations in Iquitos and Mazan districts.

| **Distrito** | **Origin** | **Destination** | **Relative importation** |
| --- | --- | --- | --- |
| **Iquitos** | Santa Rita | Iquitos | 3,691.07 |
|  | Lupuna | Iquitos | 771.96 |
|  | Santa Rita | San Pablo | 610.13 |
|  | Llanchama | Iquitos | 393.8 |
|  | San Pedro | Iquitos | 326.29 |
|  | Lupuna | Tamshiyacu | 182.67 |
|  | Santa Rita | Requena | 133.33 |
|  | Santa Rita | Santa Rosa | 91.37 |
|  | San Pedro | San Juan | 76.29 |
|  | Tarapoto | Iquitos | 49.95 |
|  | Santa Rita | Tamshiyacu | 11.73 |
|  | Lupuna | Tres Unidos | 10.62 |
|  | Tarapoto | Santo Tomas | 5.82 |
|  | San Pedro | Tamshiyacu | 5.22 |
|  | Santa Rita | Atalaya | 4.08 |
|  | Llanchama | Cacerio San Juan De Munich 9 | 3.52 |
|  |  | (Rio Itaya) |  |
|  | Tarapoto | Santa Clara | 1.78 |
|  | Tarapoto | Nuevo Milagro | 1.19 |
|  | San Pedro | Indiana | 0.82 |
|  | San Pedro | Rumococha | 0.57 |
|  | Llanchama | Santo Tomas | 0.41 |
|  | Santa Rita | Fray Martin | 0.18 |
|  | San Pedro | Tres Unidos | 0.18 |
|  | Santa Rita | Rumococha | 0.15 |
|  | Llanchama | Yarina | 0.08 |
|  | Llanchama | Zungarococha | 0.02 |
|  | Santa Rita | Manacamiri | 0.02 |
|  | Santa Rita | Padre Coca | 0.01 |
| **Mazan** | Libertad | Iquitos | 708.99 |
|  | Gamitanacocha | Iquitos | 417.88 |
|  | Huaman Urco | Iquitos | 53.81 |
|  | Salvador | Iquitos | 49.12 |
|  | Lago Yuracyacu | Iquitos | 48.92 |
|  | Libertad | Mazan | 40.9 |
|  | Libertad | Tamshiyacu | 35.74 |
|  | Libertad | Indiana | 33.51 |
|  | Salvador | Requena | 31.82 |
|  | Puerto Alegre | Iquitos | 21.06 |
|  | Gamitanacocha | Mazan | 13.99 |
|  | Huaman Urco | Indiana | 9.17 |
|  | Puerto Alegre | Tamshiyacu | 6.94 |
|  | Libertad | Santa Teresa | 5.36 |
|  | Gamitanacocha | Santa Cruz | 4.13 |
|  | Salvador | Indiana | 2.72 |
|  | Puerto Alegre | Mazan | 1.18 |
|  | Lago Yuracyacu | Indiana | 0.96 |
|  | Salvador | Mazan | 0.84 |
|  | Lago Yuracyacu | Mazan | 0.69 |
|  | Puerto Alegre | Lago Yuracyacu | 0.24 |
|  | Huaman Urco | Mazan | 0.24 |
|  | Lago Yuracyacu | Trompeteros | 0.19 |
|  | Puerto Alegre | Tipishca | 0.14 |
|  | Libertad | Santa Cruz | 0.07 |
|  | Puerto Alegre | Santa Clotilde | 0.04 |
|  | Huaman Urco | Tamanco | 0.03 |
|  | Huaman Urco | Santa Rosa | 0.01 |
|  | Lago Yuracyacu | Gamitanacocha | 0.01 |
|  | Gamitanacocha | Libertad | 0 |
