# Supplementary figures and images for: Human mobility and malaria risk in peri-urban and rural communities in the Peruvian Amazon
Source: PLoS Negl Trop Dis. 2025 Jan 6;19(1):e0012058. doi: 10.1371/journal.pntd.0012058 (PMC11737848; doi:10.1371/journal.pntd.0012058)

## Iquitos

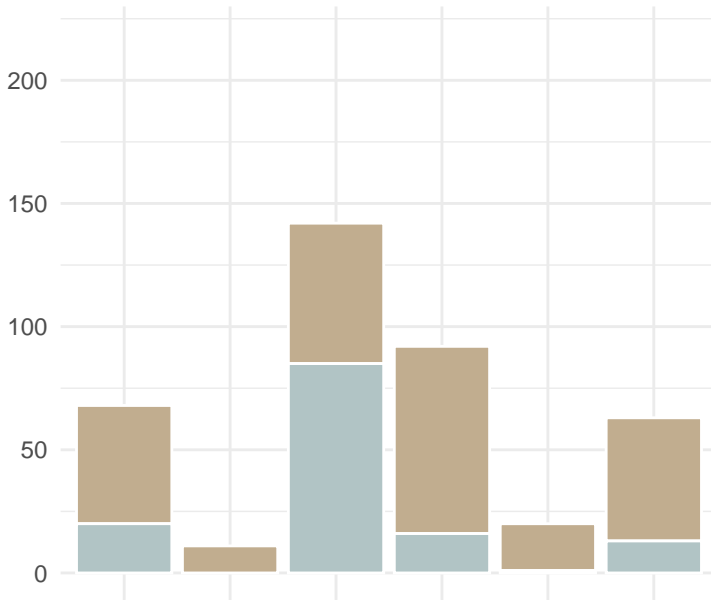

Income in the last month

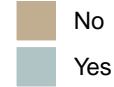

## Mazan

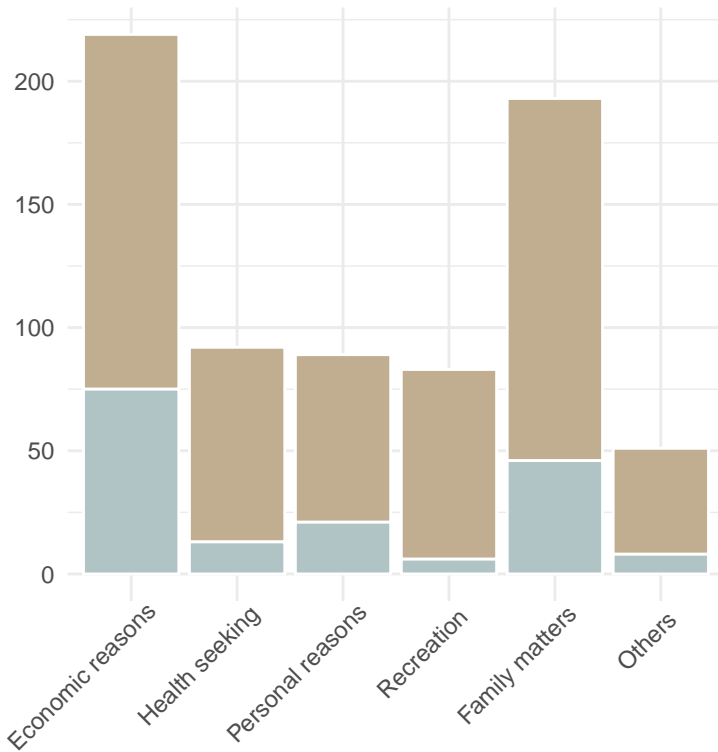

Travel reason

Supplement: S1 Fig — (PDF) [file pntd.0012058.s005.pdf]

## Iquitos

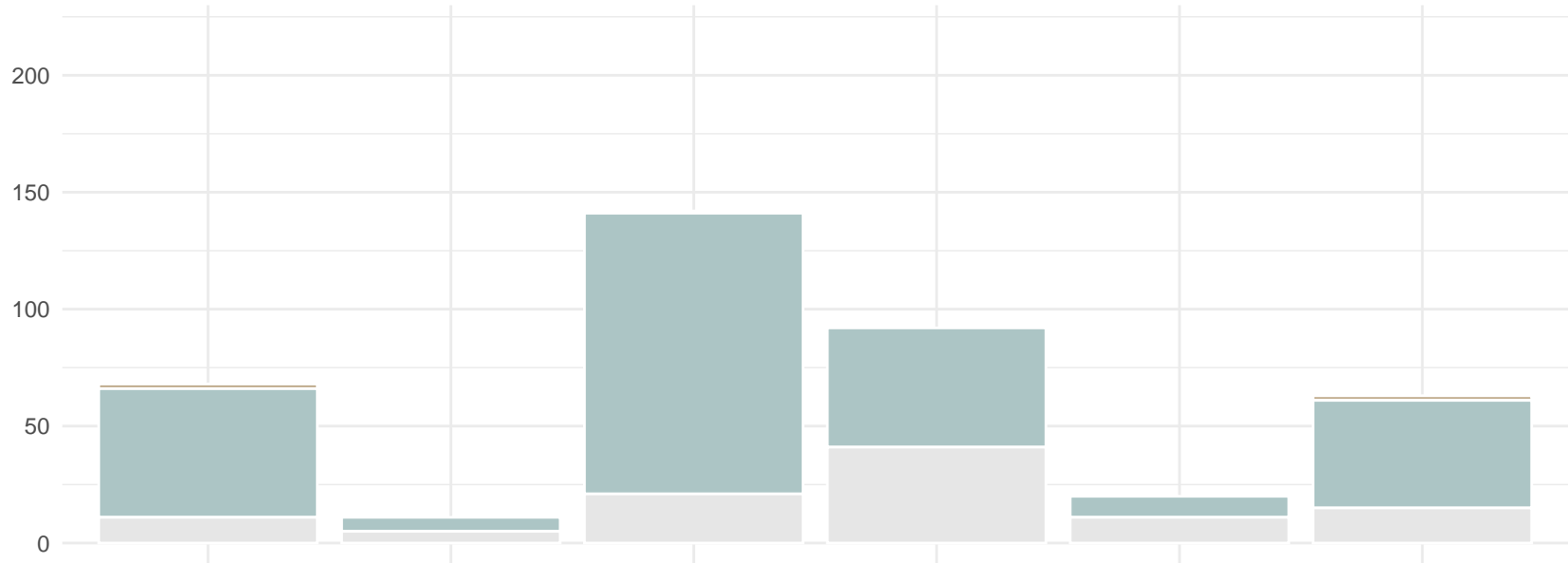

## Mazan

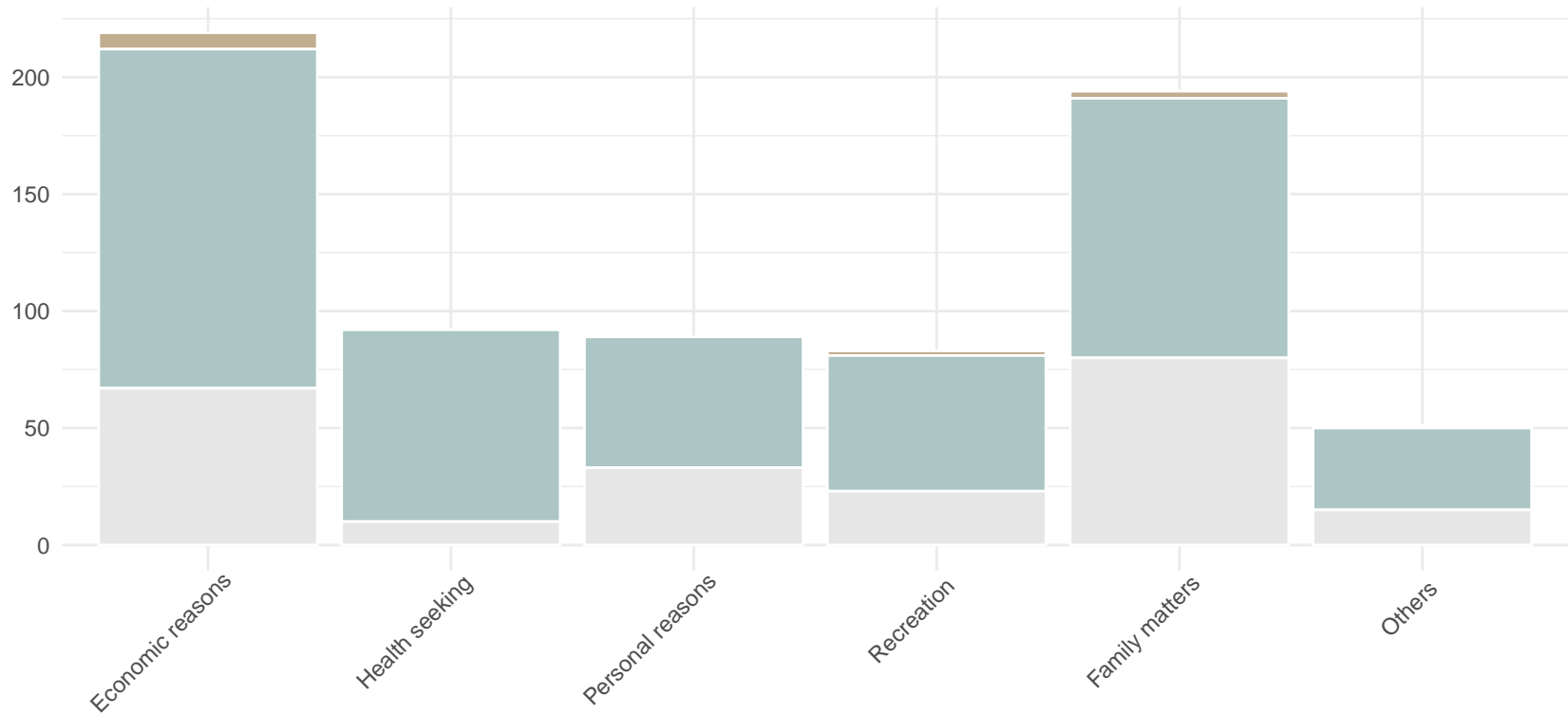

Travel reason

Time of stay

Minutes

Hours

Days

Supplement: S2 Fig — (PDF) [file pntd.0012058.s006.pdf]

A

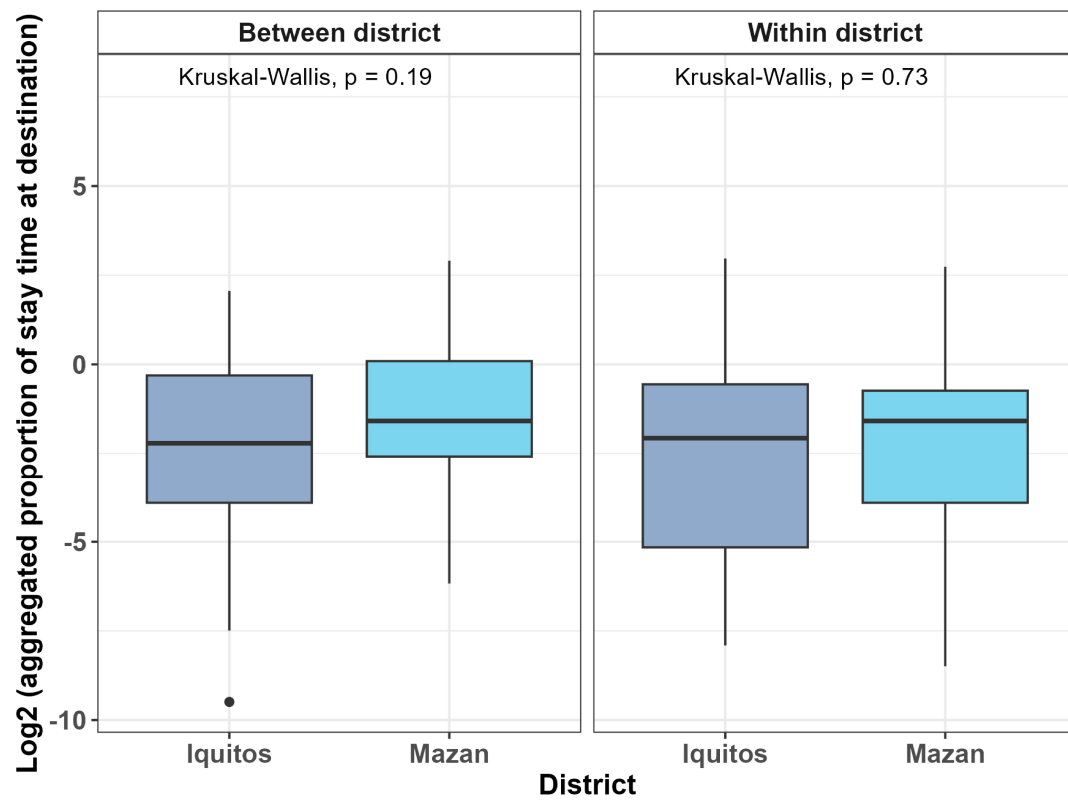

B

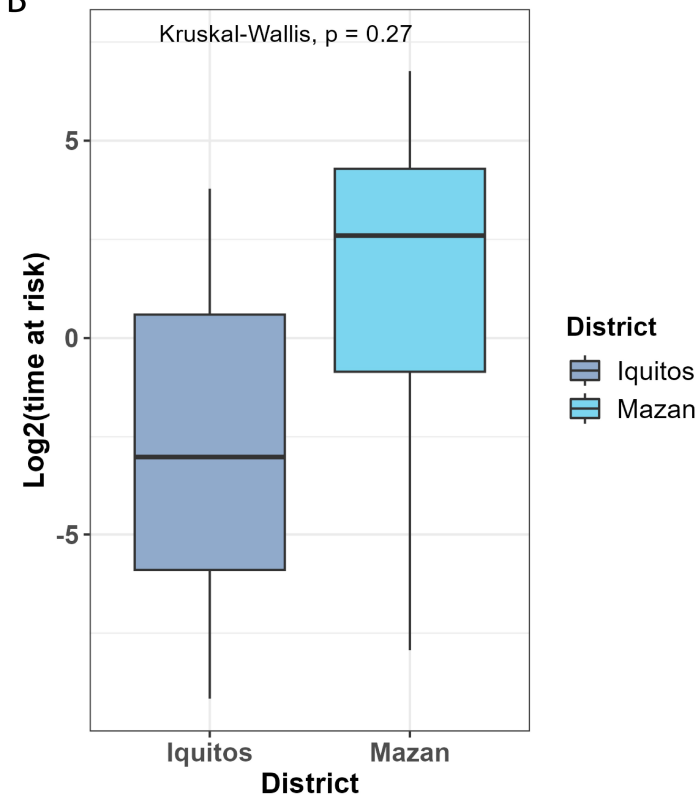

Supplement: S3 Fig — (PDF) [file pntd.0012058.s007.pdf]
