# Supplementary material for: Human mobility and malaria risk in peri-urban and rural communities in the Peruvian Amazon
Source: PLoS Negl Trop Dis. 2025 Jan 6;19(1):e0012058. doi: 10.1371/journal.pntd.0012058 (PMC11737848; doi:10.1371/journal.pntd.0012058)
Supplement: Attachment — Submitted filename: Review_PlosNTD.docx [file pntd.0012058.s009.docx]

1) Result: Social Network Analysis

It would be nice if you could provide an additional description of why comparing age against sex and travel reasons (Figures 3 and 4). I noticed that this analysis was inspired by a previous work (line 352). Is there any relation to that? If not, other demographics provided in Tables 2 and 3 can be used and compared, such as travel reasons against income or time of stay against economic activity.

2) Method: Univariate Analysis

Lines 247 and 248 mention a threshold of p-value 0.2. Please elaborate on why p-value 0.2 is used since the traditional p-value is 0.05.

3) Method: Social Network and Univariate Analysis

Could SNA and the univariate analysis be connected? It is pretty unclear to me whether the univariate (or multivariate) analysis is conducted to find significant variables before SNA or is conducted separately from SNA.
