# Supplementary material for: Human mobility and malaria risk in peri-urban and rural communities in the Peruvian Amazon
Source: PLoS Negl Trop Dis. 2025 Jan 6;19(1):e0012058. doi: 10.1371/journal.pntd.0012058 (PMC11737848; doi:10.1371/journal.pntd.0012058)
Supplement: Attachment — Submitted filename: Responses to Reviewers.docx [file pntd.0012058.s011.docx]

**Responses to Reviewer’s Questions**

Key Review Criteria Required for Acceptance? As you describe the new analyses required for acceptance, please consider the following:

**Methods**

- Are the objectives of the study clearly articulated with a clear testable hypothesis stated?
- Is the study design appropriate to address the stated objectives?
- Is the population clearly described and appropriate for the hypothesis being tested?
- Is the sample size sufficient to ensure adequate power to address the hypothesis being tested?
- Were correct statistical analysis used to support conclusions?
- Are there concerns about ethical or regulatory requirements being met?

**Reviewer #1:**

- Line 115: This section should include information about the study area. Additionally, details on the **distribution of competent malaria vectors** are necessary to draw conclusions about human mobility and malaria risks. If vector sources are **near households,** individuals may **not need to travel to contract malaria**.
  - Further details were added to the text.
- Explain the rationale behind selecting these three districts and the respective communities within each district.
  - Further details were added to the text.
- Line 134 and elsewhere: Verify the accuracy of the term “API is 60 cases per inhabitants.”
  - The Annual Parasite Index (API) is expressed in cases per population. In line 134, the correct phrase is “API is 60 cases per 1000 inhabitants”, indicating that 60 cases are expected annually for every 1000 inhabitants living in the Mazan district. Elsewhere in the manuscript, the API is no longer expressed as a measure of cases per inhabitant but as cases per location.
- Provide detailed descriptions of the data collection instrument, including their **language**, how they **were produced**, and how they **were validated**.
  - Further details were added to the manuscript.
- Although the authors are using data from a series of surveys, detailed information regarding **sample size estimation**, **inclusion** and **exclusion criteria**, and **detailed sampling procedures** for each **survey round** should be included.
  - Further details were added to the manuscript
- Line 153 to 166: Clarify the reasons for including data from two projects for the Mazan district, given that the targeted population (age, etc.) differs from the other districts. Explain how these factors were controlled for in the regression models.
  - We combined data from two projects to increase the sample size and improve model performance. In addition, the inclusion of information from the P3 project provided us with additional data on the mobility patterns of communities in peri-urban settings, allowing us to compare them with the rural scenario in the MZ district. We adjusted by the project (P1 or P3) in the multivariable analysis for Mazan to account for differences in data collection.

**Reviewer #2**: Minor revision

**Reviewer #3:** (No Response)

--------------------

**Results**

- Does the analysis presented match the analysis plan?
- Are the results clearly and completely presented?
- Are the figures (Tables, Images) of sufficient quality for clarity?

**Reviewer #1**:

- Lines 153 to 166 and Supplementary Table 1: Clarify the distinction between projects P1 and P3. P3 was conducted in all three districts among individuals over 18 years old, while P1 was only in Mazan, focusing on individuals older than three months. However, Table 2 and Table 3 include many individuals under 15 in Iquitos. Explain the categorization of age groups, particularly 15 to 40 instead of 18 to 40.
  - Further details were included in the manuscript.
  - **Table 2**: Describes the population (individuals), regardless of their sample processing. Participants without qPCR results were included in the SNA analysis, as their *Plasmodium spp.* infection is not considered when describing mobility patterns.
  - **Table 3**: Describes the population (records), regardless of their sample processing. Participants without qPCR analyses were included in part of the SNA analysis, as their *Plasmodium spp.* infection is not considered when describing mobility patterns.
- Clarify how data for certain independent variables, such as **main economic activity**, **income in the last month**, and **travel reasons**, were collected for **young children**. It seems likely that caregivers, who were also study participants, provided this information, which may affect the distribution of data in subsequent analyses. This issue also applies to results in Tables 7 and 8, where including children in variables such as **education level**, **main economic activities** (indoor or outdoor), **incomes**, and **travel-related information** could impact the associations in the regression models.
  - We updated the multivariable model and adjusted by age. The association with the main economic activity is no longer significant.
- In Tables 2 and 3, for qPCR results, were any species other than P. falciparum and P. vivax or any unidentified species detected? What does “not applicable” mean in this context?
  - Not applicable: the correct term is not available. The tables have been modified to show the correct term.
  - No other species besides *P. falciparum* and *P. vivax* were identified by molecular or microscopy analyses. Malaria transmission in the Peruvian Amazon is mainly driven by *P. vivax* and *P. falciparum.* Little to no *P. malariae* cases are reported yearly by the CDC – Peru (1).
- In Tables 7 and 8, why did the authors combine all malaria species as Plasmodium species? P. falciparum and P. vivax have different characteristics, including relapse in P. vivax, which is not associated with travel.
  - Previous studies in the Peruvian Amazon suggest species-specific heterogeneity in malaria transmission (2); however, both *P. vivax* and *P. falciparum* are transmitted by the *Ny. darlingi* vector in our area of study. Furthermore, most infections were caused by *P. vivax* in Mazan (85%, N = 69/81) and Iquitos (88%, N = 38/43). Although we could not differentiate relapses from new infections, significant associations in our model suggest that *Plasmodium spp.* infections may be related to travel events (i.e. travel to rural campsites and the number of trips).
- Lines 419 to 426 and 431 to 437: The authors reported OR values for some variables in **opposite directions**, leading to **incorrect interpretations** in the results, discussion, and conclusions. For example, in Table 7 for Mazan district, **travel in the last month is negatively associated with Plasmodium species infection**, similar to **income in the last month**. Additionally, in Iquitos, health-seeking related travel is associated with malaria infection. The authors should consider these variables to avoid misleading results and recommendations.
  - **Table 7** shows the univariate analysis of different variables and their association with *Plasmodium spp.* qPCR diagnosis. As already mentioned, traveling more than once in the last month is associated with a lower likelihood of infection compared to the reference category (Ref: One trip in the last month). These results are interpreted in lines 504-511, considering previous studies in the region. The reduced likelihood within the mobile population could be explained by the characteristics of the trip made by participants who only traveled once in the last month, with one-time travelers possibly making longer trips to rural areas than their counterparts.
  - The protective effect of health-seeking behavior observed in the Iquitos district may be due to reverse causation. Sick participants travel to seek medical care. Since health posts are more accessible in peri-urban areas, this association is only observed in communities from the IQ district and not in the MZ district.
- Line 436: Avoid using the word “surprisingly.”
  - Removed
- I noticed the authors performed a series of SNA at different stages. However, I could not find any statistical tests or SNA confirming the direct linkage between human mobility and malaria infection (even in Fig 5). I am not a demography expert, so please clarify this point.
  - Linking the SNA with the multiple regression analysis was one of the limitations of the paper. The multivariable analysis was conducted at the individual level, while the SNA analysis was at the community level. Characterization of sources and sinks is time-dependent, and extensive data would be required to get an average behavior.

Reviewer #2: Minor revision

Reviewer #3: (No Response)

--------------------

**Conclusions**

- Are the conclusions supported by the data presented?
- Are the limitations of analysis clearly described?
- Do the authors discuss how these data can be helpful to advance our understanding of the topic under study?
- Is public health relevance addressed?

Reviewer #1: (No Response)

Reviewer #2:

Reviewer #3: (No Response)

**Editorial and Data Presentation Modifications?**

Use this section for editorial suggestions as well as relatively minor modifications of existing data that would enhance clarity. If the only modifications needed are minor and/or editorial, you may wish to recommend “Minor Revision” or “Accept”.

Reviewer #1: (No Response)

Reviewer #2: Accept

Reviewer #3: (No Response)

**Result: Social Network Analysis**

- It would be nice if you could provide an additional description of why comparing age against sex and travel reasons (Figures 3 and 4). I noticed that this analysis was inspired by a previous work (line 352). Is there any relation to that? If not, other demographics provided in Tables 2 and 3 can be used and compared, such as travel reasons against income or time of stay against economic activity.
  - We conducted the suggested analysis using travel records and included a brief description in the results section.

**Method: Univariate Analysis**

- Lines 247 and 248 mention a threshold of p-value 0.2. Please elaborate on why p-value 0.2 is used since the traditional p-value is 0.05.
  - A p-value of 0.2 was used for variable pre-selection for multiple regression models. P-values <0.05 were used to determine variable significance in multiple regressions. Some variables with p-values slightly over 0.05 were kept due to relevance within the context of the study.

**Method: Social Network and Univariate Analysis**

- Could SNA and the univariate analysis be connected? It is pretty unclear to me whether the univariate (or multivariate) analysis is conducted to find significant variables before SNA or is conducted separately from SNA.
  - The univariable and multivariable analysis is conducted separately from the SNA. The SNA is an additional analysis looking to describe connectivity patterns between the communities included in our study and neighboring locations. Linking the SNA and the multivariable analysis is one of the limitations of our study, as models are at the individual level and SNA are conducted at the community level.

**Summary and General Comments**

Use this section to provide overall comments, discuss strengths/weaknesses of the study, novelty, significance, general execution and scholarship. You may also include additional comments for the author, including concerns about dual publication, research ethics, or publication ethics. If requesting major revision, please articulate the new experiments that are needed.

Reviewer #1:

**Abstract**

- Please include the main objective of the study.
- It would be beneficial to add specific numbers, percentages, or values from regression models in the results section to make the abstract more comprehensive.

**Introduction**

- The study aims to explore not only human mobility, but also other individual characteristics related to malaria risk. However, the authors have not mentioned these additional risk factors supported by other relevant studies or what new insights this study provides.
- There were some typos.
  - Further details were added to the manuscript.

**Reviewer #2**:

This is a really interesting and important analysis using a novel SNA approach to understand key drivers in P. vivax transmission related to the poorly understood and usually complex impacts of human mobility.

The analysis is robust and the results well presented. Overall the analysis would have been considerably strengthened by trying to link up parasite connectivity with the rich travel history data and SNA approach alongside an analysis of parasite genetic markers.

However the authors have appropriately acknowledged limitations of current panels for close proximity community-level differentiation and also clearly mentioned future applicability of larger highly multiplexed panels.

Otherwise I have only minor comments:

**Methods**:

- More details in methods around the conduct of the multiple surveys and how data were combined is required (in addition to the useful supplementary figure on timing/frequency). For example, were the **same households / individuals included at the multiple surveys conducted** and if yes how **was this encompassed in the results** - were they regarded as **separate individual data**, or was data **censored/excluded on subsequent surveys**, or was there any **paired/longitudinal data analysis**?  For any individual longitudinal data did travel reports vary over time i.e. seasonality for occupational or recreational activities which may impact results?
  - We tried to match records from the same individuals within the same community, but this was not part of the original P3 study design. We used personal data (names, age, sex, personal ID, birth date) to match records within the same community and project (i.e., Santa Rita, P3) and within the same community and different projects (i.e., Libertad, P1 and P3) (S1 table). However, we don’t feel comfortable doing a longitudinal analysis with this matching as it was not part of the study design, and it’s probably not fully accurate.
- It is a bit clunky although justifiable to combine cross-sectional data from 8 adult surveys (through the P3 project) across 3 districts, and child/adult surveys (through P1) from 1 district. Was there overlap in the adult populations in Mazan district between surveys?
  - It is very likely that data from P1 and P3 overlap in the adult population from the MZ district. The P3 project was designed as a series of cross-sectional screenings, while data collections from P1 considered repeated measurements. We matched data from repeated screenings in the same communities but did not explore the longitudinal variations in mobility patterns as this was not part of the original study design.
- Were the exact same questions asked by P1 and P3 surveys? i.e. harmonized (states similar structure/questions in line 180 but were differences significant enough to impact results).
  - The P1 and P3 projects were part of the NIH-*International Center for Excellence in Malaria Research* – *Amazonia* and therefore used the same questionnaire for demographic data; however, the travel-related section was extended for P1, as this was part of the project’s main aims. The same team of data collectors participated in screenings for P1 and P3. Accurate data collection was ensured through continuous training and standardized operating procedures (SOPs).
- Was the background API for San Juan comparable with Iquitos to allow this merging in the analysis?
  - In 2017, the API in the SJB district was twice the API from the IQ district (SJB API: 21.71 cases per 1,000 inhabitants; IQ API: 10.8 cases per 1,000 inhabitants) and three times in 2018 (SJB API: 33.72 cases per 1,000 inhabitants; IQ API: 11.11 cases per 1,000 inhabitants). However, the Llanchama community – the only area within SJB with data collection - is located near the enrolled communities from IQ, all accessed via the Nanay River (Fig 1). In addition, 19.4% of the processed samples in Llanchama (N: 22/113) had a positive molecular diagnosis for *Plasmodium spp*., similar to the average in IQ communities (20.66%, N: 188/618). These results suggest that malaria transmission in Llanchama resembled that of communities from IQ despite being in a different district.
- The weighting used for the SNA metrics also needs to be defined in methods. I think this is the ‘normalized number of recorded trips’ according to Fig 2 panel - good to document how this distribution was normalized. Were there any differences in outcomes based on non-weighting vs weighting?  If yes would be good to clarify and discuss what these differences mean, and if not then could consider stating this and only reporting either the raw or weighted results depending on what you think is more robust. For example, it looks like in the sub-group analyses in Fig 3 that weighted values produced more variability and differentiated e.g. female vs male outcomes in the 20-30 yr bracket.
  - Weight definitions for the SNA were included in the manuscript.
  - The edge’s weights in Fig. 2 were normalized using a modified min-max normalization method to ease visualization in the network. The min-max normalization formula was modified to obtain a minimal weight of 0.2 and a max weight of 1.
  - In SNA, weighted and non-weighted metrics express different aspects of connectivity. In this graph, the mean degree indicates the number of connections (unique destinations visited by travelers), while the mean weighted degree considers the number of trips to each destination. We present these metrics together to highlight the heterogeneity in the number of destinations (non-weighted degree) and the total visits (weighted degree across different age groups and genders.
- Could also define what in-degree and out-degree are more clearly in methods for readers not familiar with this terminology/analysis and how these are interpreted e.g. the highest degree centrality relates to the frequency of nodes being visited etc.
  - Definitions were included in the manuscript.

**Results**:

- The demographics of those enrolled looks pretty reasonable. What proportion of the adult household population were able to be enrolled?  Was there any bias here e.g. were slightly older household members or females more commonly enrolled as at home at time of survey?
  - Both P1 and P3 aimed to enroll the entire target population during each screening. If participants were not found during the first visit, the household was revisited within two days during the same screening to minimize bias during the enrolment. On average, screenings were able to enroll over 80% of the community.
- The distribution in age groups between districts varied considerably - with children <15 yrs comprising a much higher proportion of those surveyed in Mazan (46% vs 24%), which possibly had an impact on the lower proportion with a recent travel history (24%). This may be misleading as with a more representative sample recent travel in adults may not have been lower than Iquitos? You could consider looking at a separate adult only supplementary analysis to see whether these trends held (not necessarily reporting this in detail but to justify your current overall findings).
  - We replicated Table 2, table 3, univariable regression and multivariable regressions using only the adult population. The proportion of adult participants with travel history is still significantly different between Iquitos and Mazan, with a slightly higher percentage in the former (IQ: 44%, MZ: 32%). Likewise, comparisons between mobile participants from IQ and MZ (Table 3) remained similar in the adult-exclusive analysis.
  - Univariable and multivariable regressions rendered similar results. Univariable associations did not change in the adult population. The multivariable model kept the gender, number of trips in the last month and income in the last month. We included the travel destination due to its importance for our study. Odds ratios for the number of trips (Full pop. OR: 0.43; Adult pop. OR: 0.39), income in the last month (Full pop. OR: 0.28; Adult pop. OR: 0.30) and rural campsite visit (Full pop. OR: 3.46; Adult pop. OR: 3.23) were slightly affected, but the direction remained the same.
  - The main economic activity was excluded during the forward variable selection process. Possibly due to the confounding effect of age being absent after removing the youngest group. To overcome this, we updated our multivariable model (Table 8) by including age. In summary, separate adult analysis rendered similar results to the full population analysis. Confounding in the main economic activity variable was controlled by adjusting for age.
- What was classified as a main economic activity for children (for the indoor/outdoor variable given there is no N/A answer)?
  - The main economic activity question included the options “None” and “Student“. Most of the participants younger than 15 years stated being a student or having no main economic activity. However, others were involved in fishing, farming or trading activities, possibly supporting or accompanying their parents. Being a student or not having work was considered an indoor economic activity. Fishing, farming and trading were considered outdoor economic activities. We updated the multivariable model and included age to adjust for possible confounding of the association between main economic activity and *Plasmodium spp.* infection. The updated analysis revealed that the relationship between economic activity and the infection status was being confounded by age.
- There was a large proportion of those from Iquitos without qPCR results (256/939) however the trend of relatively higher malaria incidence remains robust.
  - The higher proportion of *Plasmodium spp.* infections observed in the IQ district could be caused by the conditions surrounding data collection. In communities from IQ, data collection was conducted by P3, which selected communities based on the malaria positivity index. The P3-enrolled communities had recently experienced a malaria outbreak, whereas those from P1 may not.
- Figure 5 probably not necessary in main text given no results were statistically significant here. Could consider putting this as a supplemental figure instead.
  - Moved to the supplementary figures.

**Discussion:**

- Lines 487-488 relating to reports of directional transmission of Pv need referencing and further justification i.e. how were the findings in these reports made and could they be reconsidered i.e. were they accurate in view of the data you present here.

The following citation has been included.
Llacsahuanga Allcca L, Manrique P, Delgado-Ratto C, Contreras-Mancilla J, Barbosa J, Speybroeck N, Vinetz J, Llanos-Cuentas A, Rosas-Aguirre A, Gamboa D. Dispersion pattern of Plasmodium vivax among five different settings in the Peruvian Amazon [abstract]. In: ASTMH 69th Annual Meeting; November 15-19, 2020; Virtual meeting; 2020. Abstract number 1171.

- Re factors associated with malaria positivity. Direct links with malaria prevalence and travel / human mobility still need to be considered in view of vector bionomics, prevention and access to testing/treatment. Is the same vector present across these districts (this would then effectively control for some aspects of this) and irrespective of this what may be the contribution to the spatial heterogeneity of transmission for differences in EIR, the use of preventative measures (bed nets etc) and access to testing/treatment (including radical cure) have on these analyses?
  - Malaria transmission in the Peruvian Amazon is almost exclusively driven by *Ny. darlingi.* Still, transmission across Loreto is heterogeneous, with communities within the same district showing different risk factors (3). While previous studies conducted community-specific analysis in Mazan, our sample size limited us from performing a similar analysis. To account for transmission heterogeneity, we grouped communities by district and level of rurality. Mazan communities were classified as rural, while those in Iquitos were peri-urban. Since our analysis focused on the mobile population, we expect that mobile individuals within the same district display similar mobility patterns and destinations, reducing the effect of heterogeneity in our analysis.
  - Insecticide-treated nets are distributed regularly in communities from MZ and IQ as part of Peruvian NMCPs.
- It is mentioned briefly in discussion that the vector is not present in Iquitos city. Were there any other individual data available on these metrics e.g. bed net use which could have been added to the uni- and multi-variate model? Results here are also **more difficult to interpret** given the **relapsing nature of liver stage P. vivax** infections which would not be due to travel-related causes - possibly worth mentioning briefly.
  - The absence of vectors in the city of Iquitos was discussed in the context of the parasite connectivity results. Due to the high frequency of travel to Iquitos city, our parasite connectivity index suggested it as a sink for malaria parasites. However, *Ny. darlingi* mosquitoes are present in communities surrounding the Iquitos city (20-60 km) and are rarely reported within the city(4). Due to the high incidence of dengue, bed net use is common in the city of Iquitos; however, our dataset does not include data collection in the city, but rather in the surroundings areas (peri-urban communities).

**Note**: The relapsing nature of *P.vivax* was mentioned in other comments. I will include in the discussion, as one of our limitations, that we cannot differentiate between relapses and new infections.

- The finding that frequent travel is negatively associated with malaria prevalence is interesting as is the justification in discussion that this may be attributable to people with extended duration work related travel rather than frequent travel being at higher exposure risk. Wasn’t travel duration data available and could this be analyzed in the model to try and evaluate this hypothesis?

Questions regarding the duration of the last trip were not included in the P3 project questionnaires, as it was not part of their main aims. Therefore, we cannot include that variable in the model without discarding a significant portion of our dataset. We suggest that future studies focus on a design that captures high-resolution data on mobile populations, such as weekly active case detection (ACD) or individual GPS tracking.
